# Adherence to a Western dietary pattern and risk of bladder cancer: A pooled analysis of 13 cohort studies of the Bladder Cancer Epidemiology and Nutritional Determinants international study

**DOI:** 10.1002/ijc.33173

**Published:** 2020-07-20

**Authors:** Mostafa Dianatinasab, Anke Wesselius, Amin Salehi‐Abargouei, Evan Y. W. Yu, Maree Brinkman, Mohammad Fararouei, Piet van den Brandt, Emily White, Elisabete Weiderpass, Florence Le Calvez‐Kelm, Marc Gunter, Inge Huybrechts, Fredrik Liedberg, Guri Skeie, Anne Tjonneland, Elio Riboli, Graham G. Giles, Roger L. Milne, Maurice P. Zeegers

**Affiliations:** ^1^ Center for Health Related Social and Behavioral Sciences Research Shahroud University of Medical Sciences Shahroud Iran; ^2^ Department of Complex Genetics and Epidemiology, School of Nutrition and Translational Research in Metabolism Maastricht University Maastricht The Netherlands; ^3^ Nutrition and food security research center, Department of Nutrition, School of Public Health Shahid Sadoughi University of Medical Sciences Yazd Iran; ^4^ Department of Clinical Studies and Nutritional Epidemiology Nutrition Biomed Research Institute Melbourne Victoria Australia; ^5^ Cancer Epidemiology Division Cancer Council Victoria Melbourne Victoria Australia; ^6^ Department of Epidemiology Shiraz University of Medical Sciences Shiraz Iran; ^7^ Department of Epidemiology, Schools for Oncology and Developmental Biology and Public Health and Primary Care Maastricht University Medical Centre Maastricht The Netherlands; ^8^ Fred Hutchinson Cancer Research Center Seattle Washington USA; ^9^ International Agency for Research on Cancer World Health Organization Lyon France; ^10^ Department of Urology Skåne University Hospital Malmö Sweden; ^11^ Institution of Translational Medicine Lund University Malmö Sweden; ^12^ Department of Community Medicine UIT The Arctic University of Norway Tromsø Norway; ^13^ Danish Cancer Society Research Center Copenhagen Denmark; ^14^ Department of Public Health University of Copenhagen Copenhagen Denmark; ^15^ Department of Epidemiology and Biostatistics, School of Public Health Imperial College London London UK; ^16^ Centre for Epidemiology and Biostatistics Melbourne School of Population and Global Health, The University of Melbourne, 207 Bouverie Street Melbourne Victoria Australia; ^17^ Precision Medicine, School of Clinical Sciences at Monash Health Monash University Clayton Victoria Australia; ^18^ CAPHRI School for Public Health and Primary Care Maastricht University The Netherlands; ^19^ School of Cancer Sciences University of Birmingham Birmingham UK

**Keywords:** bladder cancer, epidemiology, risk factor, Western diet

## Abstract

Little is known about the association of diet with risk of bladder cancer. This might be due to the fact that the majority of studies have focused on single food items, rather than dietary patterns, which may better capture any influence of diet on bladder cancer risk. We aimed to investigate the association between a measure of Western dietary pattern and bladder cancer risk. Associations between adherence to a Western dietary pattern and risk of developing bladder cancer were assessed by pooling data from 13 prospective cohort studies in the “BLadder cancer Epidemiology and Nutritional Determinants” (BLEND) study and applying Cox regression analysis. Dietary data from 580 768 study participants, including 3401 incident cases, and 577 367 noncases were analyzed. A direct and significant association was observed between higher adherence to a Western dietary pattern and risk of bladder cancer (hazard ratio (HR) comparing highest with lowest tertile scores: 1.54, 95% confidence interval (CI): 1.37, 1.72; *P*‐trend = .001). This association was observed for men (HR comparing highest with lowest tertile scores: 1.72; 95% CI: 1.51, 1.96; *P*‐trend = .001), but not women (*P*‐het = .001). Results were consistent with HR above 1.00 after stratification on cancer subtypes (nonmuscle‐invasive and muscle‐invasive bladder cancer). We found evidence that adherence to a Western dietary pattern is associated with an increased risk of bladder cancer for men but not women.

## Abstract 2

**What's new?**

Does diet affect bladder‐cancer risk? Individual foods are rarely eaten in isolation, but little is known about the impact of overall dietary habits. In this large, prospective study, the authors found that greater adherence to a Western dietary pattern was associated with a significantly increased risk of bladder cancer in men. (Surprisingly, the same effect was not seen in women.) Further research is needed to identify the specific food types responsible and their mechanisms of bladder carcinogenesis. However, education to encourage changes in general dietary habits may provide a valuable public‐health benefit.

**Abbreviations**
BLEND: BLadder cancer Epidemiology and Nutritional Determinants
BMI: body mass index
CIs: confidence intervals
DPs: dietary patterns
FFQ: food frequency questionnaire
HRs: hazard ratios
HCAs: heterocyclic amines
MIBC: muscle‐invasive bladder cancer
NMIBC: nonmuscle‐invasive bladder cancer
PAHs: polycyclic aromatic hydrocarbons
RR: relative risk
WDS: Western diet score

## 1 INTRODUCTION

Recent estimates from the International Agency for Research on Cancer (IARC) rank bladder cancer globally as the seventh and seventeenth most common malignancy for men and women, respectively.^1^, ^2^ Most (75%) cancers are nonmuscle‐invasive bladder cancer (NMIBC) that frequently recur but require intensive treatment and follow‐up measures posing a large burden on national health care budgets and patient quality of life.^2^, ^3^

Epidemiological studies have identified several factors which potentially influence bladder cancer risk, including; sex, smoking, age and occupation.^3^, ^4^, ^5^ In addition, evidence suggests that other factors related to environmental and lifestyle (eg, body mass index [BMI], physical activity and diet) also might affect the bladder cancer risk.^6^, ^7^ Since the bladder is an excretory organ, diet might especially play an essential role in the development of bladder cancer.^8^ Previous research reported that high fluid, fruit, vegetable and yogurt intakes are associated with a reduced risk,^9^ while barbecued meat, pork and total fat intakes are associated with an increased risk.^10^, ^11^, ^12^

Nutritional observational studies have long focused on associations between single food items and disease risk. However, given that individuals do not consume foods (or nutrients) in isolation, but in a complex combination of multiple foods (or nutrients), this single food item approach might be unable to measure the impact of the interaction among different foods on disease risk. Therefore, an increasing number of researchers are taking a more holistic dietary approach, by defining food consumption patterns to characterize a population's dietary intake and to examine potential relationships of these patterns with disease risk. However, although this approach has received much attention during the past few years, evidence on the relation between dietary patterns (DPs) and bladder cancer risk remains scarce. As a consequence of the Neolithic‐ and Industrial revolutions, which introduced staple foods and new methods of food processing, the Western diet was introduced.^13^ The Western dietary pattern is characterized by high intakes of red and processed meat, fast foods, convenience products, sugary soft drinks, snacks, eggs, refined cereals, high‐fat dairy products and hydrogenated fat.^14^, ^15^, ^16^, ^17^ Particularly meats, eggs and dairy products are considered as prominent features of the Western diet.^18^, ^19^, ^20^ This dietary pattern has been linked to a range of health outcomes, including several types of cancer. Evidence for any association between a Western dietary pattern and bladder cancer risk is limited. To the best of our knowledge, only one study has investigated this association. In a multi‐centric, hospital‐based, case‐control study in Montevideo, Uruguay, it was found that people who adhered to a Westernized diet had 2.35 times higher risk of bladder cancer.^21^

Given the biases to which case‐control studies are prone, we aimed to investigate prospectively the potential association between adherence to a Western dietary pattern and the risk of bladder cancer, by pooling data from 13 prospective cohort studies in the BLEND consortium.

## 2 METHODS

### 2.1 Study sample

The study was conducted within the of the BLEND consortium. BLEND is a large international nutritional consortium, which includes 16 prospective cohort studies from several populations.^22^ For the current study, data from 13 cohorts with sufficient collected information on the intake of food items of interest (ie, those required for scoring the chosen Western dietary pattern) were included in the analyses. Studies originated from centers in Australia,^23^, ^24^ Denmark,^25^ France,^26^ Germany,^27^ Greece,^28^ Italy,^29^ Norway,^30^ Spain,^28^ Sweden,^31^, ^32^ the Netherlands,^33^, ^34^ the United Kingdom,^35^, ^36^ and the United States.^37^

### 2.2 Data collection and coding

Details of BLEND consortium protocols and methodology have been described elsewhere.^22^ Briefly, the primary data from all included studies were gathered into an integrated database. Data were checked and the food consumption was converted to grams per day by the use of country‐specific food tables and the frequency responses. Each study ascertained incident bladder cancer, defined to include all urinary bladder neoplasms according to the International Classification of Diseases for Oncology (ICD‐O‐3 code C67) using population‐based cancer registries, health insurance records or medical records.^38^

Dietary data were obtained using a validated food frequency questionnaires (FFQ), and were recorded using the Eurocode 2 food coding system.^39^ In addition to the information on dietary intake, other baseline data included study characteristics, for example, design, method of dietary assessment, recall period of dietary intake and geographical region, demographic information (age, sex and ethnicity), pathology of bladder cancer (disease subtype; nonmuscle‐invasive bladder cancer [NMIBC] and muscle‐invasive bladder cancer [MIBC]) and smoking status (current/former/never) and quantity (packs/year), all measured at baseline.

### 2.3 Western diet score

In the present study, eight food groups were selected to define the Western dietary pattern. This selection was based on prior knowledge^14^, ^15^, ^16^, ^17^, ^18^, ^19^, ^20^ and data availability and included eggs, butter, margarine, animal fat, sugar and sugar added products, red and processed meats, dressings, and dips. For each food item, a score from 1 to 5 was assigned based on quintiles of overall intake. A score of “1” was assigned to those in the lowest quintiles and “5” was assigned to those in the highest quintiles. Each participant's overall score was calculated by summing the scores received for each individual food item. Accordingly, the score ranged from 8 (minimal adherence) to 40 (highest adherence). Participants were then classified into tertiles (low, medium and high adherence to a Western dietary pattern) according to their score.

### 2.4 Statistical analysis

Baseline characteristics of study participants were compared between the tertiles of adherence to the Western dietary pattern using analysis of variance or independent sample *t* test, for continuous variables or ANCOVA for categorical variables. We used the Cox proportional hazard modeling approach with recruitment as the starting point on the time scale to assess the association between adherence to the Western dietary pattern and bladder cancer risk. Hazard ratios and 95% confidence intervals (CIs) for developing bladder cancer were calculated with the first tertile assigned as a reference group. The proportional hazards assumption was examined graphically and we found no apparent violation of the assumption. Survival time was estimated by subtracting age at exit by age at entry in the cohort as T0, thereby correcting for age in the analysis. Study was included as a random effect. The Cox regression models were performed as crude, and adjusted Model 1 for: total energy intake in kilocalories, sex, smoking status (never, former or current smoker) and smoking intensity ([pack/day] * years), and additionally for fluid, vegetables and fruits intake (Model 2). Analyses were stratified on smoking status, sex and disease subtype (nonmuscle‐invasive or muscle‐invasive disease). All statistical analyses were performed using Stata/SE version 14.2. *P* values less than .05 were considered as statistically significant.

## 3 RESULTS

### 3.1 Baseline characteristics

Dietary data from 580 768 study participants, including 3401 incident cases and 577 367 noncases were analyzed, with a total of 6 451 306 person‐years of follow‐up (median follow‐up: 11.4 years). Disease type was known for 2570 cases, of which 945 (36.7%) were MIBC and 1625 (63.3%) were NMIBC. Baseline characteristics of the study sample are presented in Table 1.

**TABLE 1:** General characteristics of participants by cohort study

Characteristics	NLCS^33^ (n = 5238)	VITAL^37^ (n = 66 518)	CVV and MCCS (n = 37 218)	EPIC‐Denmark^25^ (n = 55 670)	EPIC‐France^26^ (n = 64 204)	EPIC‐Germany^27^ (n = 48 754)	EPIC‐Greece (n = 25 005)	EPIC‐Italy^29^ (n = 44 663)	EPIC‐Spain (n = 40 389)	EPIC‐Sweden (n = 48 625)	EPIC‐the Netherlands^34^ (n = 36 801)	EPIC‐the UK (n = 74 379)	EPIC‐Norway (n = 33 304)	Total (n = 580 768)
Subjects (n)														
Case/noncase	876/4362	337/66 181	503/36 715	386/55 284	31/64 173	205/48 549	50/24 955	186/44 477	149/40 240	301/48 324	107/36 694	247/74 132	23/33 281	3401/577 367
Person‐year	73 688.8	448 995.4	715 158.9	608 813	667 809.9	482 453.3	238 122	502 020.3	487 491.1	638 482.8	434 974.5	828 991.7	6 437 305.7	6 451 306
Baseline age (mean ± SD)														
Case	62.73 (4.09)	66.16 (7.01)	59.90 (7.37)	58.50 (4.37)	58.04 (6.00)	56.41 (7.13)	60.89 (10.31)	55.24 (6.75)	54.49 (7.19)	60.27 (7.07)	56.20 (8.03)	63.62 (9.98)	49.30 (4.38)	60.50 (7.35)
Noncase	61.85 (4.21)	61.18 (7.37)	54.96 (8.67)	56.67 (4.37)	52.74 (6.63)	50.55 (8.56)	53.30 (12.59)	50.50 (7.92)	49.19 (8.03)	51.93 (10.89)	48.94 (11.93)	49.05 (14.34)	48.07 (4.30)	52.66 (10.14)
Sex *n* (%)														
Men	2867 (54.73)	33 394 (50.20)	15 267 (41.02)	26 532 (47.66)	0 (0.00)	21 168 (43.42)	10 327 (41.30)	13 774 (30.84)	15 259 (37.78)	22 214 (45.68)	9629 (26.17)	22 260 (29.93)	0 (0.00)	192 691 (33.18)
Women	2371 (45.27)	33 124 (49.80)	21 951 (58.98)	29 138 (52.34)	64 204 (100.00)	27 586 (56.58)	14 678 (58.70)	30 889 (69.16)	25 130 (62.22)	26 411 (54.32)	27 172 (73.83)	52 119 (70.07)	33 304 (100.00)	388 077 (66.82)
Smoking status n (%)														
Current smoker	1613 (30.79)	5366 (8.07)	4164 (11.19)	19 140 (34.38)	5862 (9.13)	10 165 (20.85)	6899 (27.59)	12 385 (27.73)	10 847 (26.86)	11 474 (23.60)	11 233 (30.52)	9040 (12.15)	11 101 (33.33)	119 289 (20.54)
Former smoker	1930 (36.85)	29 644 (44.57)	11 576 (31.10)	16 998 (30.53)	13 013 (20.27)	16 194 (33.22)	4195 (16.78)	11 945 (26.74)	7147 (17.70)	13 269 (27.29)	11 501 (31.25)	23 724 (31.90)	10 292 (30.90)	171 428 (29.52)
Never smoker	1695 (32.36)	31 508 (47.37)	21 478 (57.71)	19 532 (35.09)	45 329 (70.60)	22 395 (45.93)	13 911 (55.63)	20 333 (45.53)	22 395 (55.45)	23 882 (49.11)	14 067 (38.22)	41 615 (55.95)	11 911 (35.76)	290 051 (49.94)
Smoking intensity pack‐year (mean ± SD)^a^	32.89 (12.28)	26.25 (23.49)	25.01 (13.03)	19.73 (17.74)	22.52 (16.66)	11.32 (13.47)	10.96 (14.85)	12.83 (14.02)	10.57 (13.70)	12.26 (15.09)	14.28 (14.81)	8.51 (13.30)	14.01 (13.47)	17.01 (15.07)

^a^ Among past and current smokers; pack‐years = number of packs of cigarettes smoked per day multiplied by the number of years of smoking.

In total, 192 691 (33%) men and 388 077 (67%) women were included. As shown in Table 1, compared to noncases, bladder cancer cases were more likely to be men (76%) and to be current (36%) or former smokers (43%). Mean (±SD) age was 52.7 years (±10.2) for cases and 60.5 (±7.3) 52.6 (±10.1) for controls. The median (interquartile) time from exposure collection to diagnosis with bladder cancer was 8.5 years (4.9‐12.0).

Baseline characteristics and dietary information based on tertiles of adherence to the Western dietary pattern are reported in Table 2. Roughly 1264 (37%) of the cases were in the highest tertile of adherence to the Western dietary pattern compared to 184 291 (32%) for noncases. Current smokers with a high smoking intensity were more common among those in the highest tertile of adherence to the Western dietary pattern (39%) compared to those in lower tertiles of adherence (28%). The mean (±SD) of the WDS was 23.1 (4.2) and 22.3 (4.5) for cases and noncases, respectively.

**TABLE 2:** Baseline characteristics and dietary items based on participants’ status and Western diet score tertile

	Participants^a^			WDS tertile^b^			
Characteristics	Cases	Noncases	*P* value	Tertile 1	Tertile 2	Tertile 3	*P* value
Participants (n (%))							
Case/noncase	—	—	—	822 (24.16)/198 253 (34.34)	1315 (38.67)/194 823 (33.74)	1264 (37.17)/184 291 (31.92)	<.001^c^
Person‐year	28 455.67	6 422 851	<.001^d^	2 086 731	2 243 150	2 121 425	<.001^e^
Baseline age (mean ± SD)	60.50 (7.35)	52.66 (10.14)	<.001^d^	53.87869 (10.39)	52.28 (10.44)	51.91639 (9.42)	<.001^e^
WD score (mean ± SD)	23.05 (4.21)	22.30 (4.51)	.001	17.44 (2.27)	22.37 (1.13)	27.46 (2.19)	.001^e^
Cancer subtype (n (%))							
NMIBC	1365	—	—	334 (24.47)	547 (40.07)	484 (35.46)	.184^c^
MIBC	874	—		189 (21.62)	380 (43.48)	305 (34.90)	
Sex n (%)							
Men	2579 (75.83)	190 112 (32.93)	<.001^c^	58 159 (30.18)	63 315 (32.86)	71 217 (36.96)	<.001^c^
Women	822 (24.17)	387 255 (67.07)		140 916 (36.31)	132 823 (34.23)	114 338 (29.46)	
Smoking status n (%)							
Current smoker	1235 (36.31)	118 054 (20.45)	<.001^c^	33 360 (27.97)	39 344 (32.98)	46 585 (39.05)	<.001^c^
Former smoker	1462 (42.99)	169 966 (29.44)		60 750 (35.44)	57 471 (33.52)	53 207 (31.04)	
Never smoker	704 (20.70)	289 347 (50.11)		104 965 (36.19)	99 323 (34.24)	85 763 (29.57)	
Smoking intensity pack‐year (mean ± SD)	33.33 (12.71)	23.61 (12.47)	<.001^d^	22.20 (12.52)	23.49 (12.48)	25.00 (12.39)	<.0001^e^
Cream gram per day (mean ± SD)	2.13 (7.32)	2.33 (4.72)	.01^d^	1.55 (3.87)	2.36 (4.69)	3.14 (5.47)	<.0001^e^
Egg gram per day (mean ± SD)	17.84 (15.19)	16.96 (16.09)	.001^d^	10.25 (11.21)	16.29 (14.58)	24.90 (18.40)	<.0001^e^
Red and processed meet gram per day (mean ± SD)	92.75 (58.72)	78.85 (60.78)	<.001^d^	48.21 (42.30)	73.05 (54.61)	118.11 (62.51)	<.0001^e^
Butter gram per day (mean ± SD)	5.08 (10.97)	3.84 (8.18)	<.001^d^	1.80 (5.51)	3.74 (8.01)	6.18 (9.99)	<.0001^e^
Margarine gram per day (mean ± SD)	18.26 (20.20)	11.28 (15.46)	<.001^d^	7.84 (12.93)	11.51 (15.41)	14.85 (17.20)	.001^e^
Animal fat gram per day (mean ± SD)	0.22 (1.54)	0.21 (1.16)	.35^d^	0.02 (0.29)	0.11 (0.91)	0.51 (1.78)	<.0001^e^
Pasta gram per day (mean ± SD)	32.04 (48.63)	35.31 (50.49)	.001^d^	32.43 (39.75)	32.22 (42.10)	41.62 (65.94)	<.0001^e^
Sugar gram per day (mean ± SD)	16.70 (21.01)	18.02 (47.57)	.10^d^	10.94 (26.97)	15.81 (43.24)	27.92 (64.32)	<.0001^e^
Dressing gram per day (mean ± SD)	4.79 (7.44)	6.30 (9.83)	<.001^d^	2.80 (6.61)	6.24 (9.66)	10.08 (11.36)	<.0001^e^
Dips gram per day (mean ± SD)	4.41 (9.47)	5.57 (9.57)	<.001^d^	2.99 (6.26)	5.85 (9.06)	8.01 (12.03)	<.0001^e^
Vegetables gram per day (mean ± SD)	206.92 (138.40)	198.94 (141.96)	<.001^d^	184.04 (150.51)	204.76 (141.26)	208.91 (131.48)	<.0001^e^
Fruits gram per day (mean ± SD)	122.53 (111.26)	120.33 (110.10)	.24^d^	109.94 (111.78)	122.03 (106.63)	129.71 (110.95)	<.0001^e^
Fluid milliliters per day (mean ± SD)	1563.81 (861.36)	1429.51 (878.16)	.001^d^	1244.57 (786.36)	1427.15 (817.62)	1632.87 (982.52)	<.001^e^

Abbreviations: MIBC, muscle‐invasive bladder cancer; NMIBC, nonmuscle‐invasive bladder cancer; WDS, Western diet score.

^a^ 100 % is computed across column (participants’ status).

^b^ 100% is computed across rows (study variables).

^c^ Based on ANCOVA.

^d^ Based on independent sample t‐test.

^e^ Based on one‐way analysis of variance.

### 3.2 Associations between the Western dietary pattern and bladder cancer risk

The HR estimates for bladder cancer associated with adherence to the Western dietary pattern are presented in Table 3. Overall, greater adherence to the Western dietary pattern was associated with an increased risk of bladder cancer (Model 2: HR comparing highest with the lowest tertile: 1.54, 95% CI: 1.37, 1.72). Test for linear trend across the tertiles of Western dietary pattern adherence was significant (*P*‐trend = .001). Results for men (Model 2: HR highest compared to lowest tertile: 1.72, 95% CI: 1.51, 1.96 (*P*‐trend = .001) were comparable and in line with the overall estimates. For women, no evidence of association (Model 2: HR highest compared to lowest tertile: 1.09, 95% CI: 0.86, 1.38) was observed (*P*‐trend = .46; *P*‐het = .001).

**TABLE 3:** Hazard ration (HR) and 95% confidence intervals (CIs) based on tertile of Western diet score

	Tertile 1 HR (95% CI) 18 (16, 19)^a^	Tertile 2 HR (95% CI) 22 (21, 23)^a^	Tertile 3 HR (95% CI) 27 (26, 29)^a^	*P* trend
All participants				
Participants (n) Case/noncase	822/198 253	1315/194 823	1264/184 291	—
Pearson year	2 086 731	2 243 150	2 121 425	—
Crude	1 (reference)	1.51 (1.38, 1.65)	1.76 (1.61, 1.92)	<.001
Model 1^b^	1 (reference)	1.30 (1.18, 1.43)	1.33 (1.20, 1.48)	<.001
Model 2^c^	1 (reference)	1.44 (1.29, 1.59)	1.54 (1.37, 1.72)	.001
Women				—
Participants (n) Case/noncase	258/140658	342/132481	222/114116	—
Pearson year	1 508 860	1 519 577	1 298 213	—
Crude	1 (reference)	1.30 (1.11, 1.53)	1.10 (0.91, 1.31)	.213
Model 1^b^	1 (reference)	1.24 (1.01, 1.52)	1.06 (0.85, 1.34)	.584
Model 2^c^	1 (reference)	1.25 (1.02, 1.54)	1.09 (0.86, 1.38)	.466
Men				
Participants (n) Case/noncase	564/57595	973/62342	1042/70175	—
Pearson year	577 871.8	723 572.9	823 212.2	—
Crude	1 (reference)	1.50 (1.35, 1.67)	1.68 (1.51, 1.86)	.001
Model 1^b^	1 (reference)	1.33 (1.19, 1.48)	1.42 (1.26, 1.59)	.001
Model 2^c^	1 (reference)	1.53 (1.35, 1.73)	1.72 (1.51, 1.96)	.001

Abbreviations: CI, confidence interval; HR, hazard ratio.

^a^ Median WD score (range).

^b^ Adjusted for energy intake, smoking status, smoking intensity, age and sex.

^c^ Adjusted for Model 1+ fluid intake, fruit and vegetable intakes.

After stratification by sex and smoking the findings were in line with the overall results suggesting that apart from smoking status, higher adherence to the Western diet is a risk factor for men but not women (Table S1). Additionally, after stratification by disease subtype, results remained consistently above 1.00 for both NMIBC (HR: 1.28, 95% CI: 1.02, 1.63) and MIBC (HR: 1.28, 95% CI: 1.01, 1.64) patients (Table S2).

In the present study, it was also assessed whether any association with the Western dietary pattern would change by excluding each single component of the Western diet. Results, however, remained stable and therefore are not reported.

## 4 DISCUSSION

Using prospective cohort studies data from the BLEND consortium, we investigated associations between adherence to a Western dietary pattern and bladder cancer risk and observed an overall direct association between a high adherence to Western dietary pattern and bladder cancer risk for men, but not women. Analyses stratified by disease subtype showed similar results to the overall findings, indicating that the association is unlikely to be confounded by factors that might differ between the different bladder cancer subtypes.

Although we are the first to examine an a priori defined Western dietary pattern in association with bladder cancer risk, a previous study, identified a factor analysis derived Western dietary pattern to be associated with bladder cancer risk.^21^ De‐Stephani et al suggested that adherence to a Western dietary pattern is associated with a 2.3‐fold risk of bladder cancer. Similar results were reported for bladder cancer recurrence, with individuals who highly adhere to the Western dietary pattern experiencing a 1.48 times higher risk of recurrence compared to those with low adherence to the Western dietary pattern.

Although evidence of association for the whole Western dietary pattern with bladder cancer risk is limited, several studies have focused on some key elements of this dietary pattern and reported positive associations. Red and processed meat is such an element positively associated with bladder cancer risk. A recent meta‐analysis showed, by combining results from five cohort studies and eight case‐control studies, an increment of 50 g of processed meat per day was associated with 20% increased risk of bladder cancer.^40^ In addition, the authors showed that red meat consumption was associated with bladder cancer, with a 51% increased risk per increment of 100 g per day. However, this association with red meat consumption could only be observed among case‐control studies. More recently, this association was confirmed by a cohort study.^41^ The effect of meat consumption may be explained by the carcinogenic compounds that are produced during the cooking and processing of meat, which includes nitrate, nitrite, heterocyclic amines and polycyclic aromatic hydrocarbons. Since these compounds are excreted in the urine, they come in close contact with the inner lining of the bladder wall which may exert a carcinogenic effect on urothelial cells.

Another element of the Western dietary pattern that might explain the adverse effect of this diet on bladder cancer risk is fat intake.^10^, ^42^, ^43^ A meta‐analysis conducted in 2000 by Steinmaus et al,^44^ found that high fat intake significantly elevated the risk of bladder cancer (relative risk [RR] = 1.37, 95% CI: 1.16, 1.62). This was confirmed by the Netherlands Cohort Study on diet and cancer that reported that a high intake of butter increased bladder cancer risk by 61%.^45^ In contrast, a Japanese cohort study could not find an association between butter intake and bladder cancer risk.^46^ In line with these findings, a Belgian case‐control study could not detect any association between high intake of animal products, which are also high in their fat content, and bladder cancer risk.^47^ More research on fat consumption, and the different sources of fat, is needed to elucidate any role of fat intake and different sources of fat on bladder cancer risk.

Eggs contain a lot of cholesterol, which has been shown to increase the formation of secondary bile acids in both humans and animals. Bile acids are linked to several mechanisms causing cancer.^48^ In addition, eggs can also be a source of heterocyclic amines when cooked in high temperatures.^49^ A meta‐analysis, including four cohort studies and nine case‐control studies, however, did not observe an association between egg consumption and bladder cancer risk, except for a possible positive relationship with the intake of fried eggs.^50^ It, therefore, remains inconclusive whether egg intake contributes to the positive association of the Western dietary pattern with bladder cancer risk identified in our study.

Sugar is another important element of the Western dietary pattern that has been investigated but its influence on risk of bladder cancer remains inconclusive. While the NIH‐AARP Diet and Health Study showed that sugar is not significantly associated with the risk of bladder cancer,^51^ Stefani et al,^21^ showed that sugar intake may increases the risk of bladder cancer by 124%. When studying sweetened beverages, which are considered the main sugar source, results are more in line, in that regular consumption is positively associated with bladder cancer risk.^52^, ^53^ Unfortunately, due to lack of data, we were unable to include sugar‐sweetened beverages in our Western dietary pattern analysis, which might have led to underestimation of our result.

In the present study, the sex‐stratified results showed a diversity (*P*‐het = .001) in the association between high adherence to the Western dietary pattern and the risk of bladder cancer for men and women. An explanation for this observation might be genetic variability by sex, which might cause a different effect of similar environmental exposures to the bladder carcinogenesis.^54^, ^55^ It has been suggested that gender disparity in bladder cancer risk could be explained by sex‐specific differences in the metabolism of bladder cancer carcinogens that are influenced by sex hormone.^56^ However, the mechanisms by which Western diet could modulate bladder cancer risk differently in men and women remain to be explored. Furthermore, the limited number of women cases (n = 822) could also affect the outcome of the analyses. Research on the epigenetics of diet and bladder cancer remains in its infancy and need to be explored in detail in future research. Results of the sex and smoking stratified analyses showed no difference between smokers and nonsmokers. Therefore, the effect of residual confounding of smoking on the relation between the Western diet and bladder cancer is suggested to be minor. Finally, to determine the single study effect, sensitivity analyses were performed by removing each individual study in turn from the main analysis. Results showed that the main finding remained robust.

### 4.1 Strengths and limitations

Although BLEND is so far the largest pooled cohort study investigating the associations between adherence to a Western dietary pattern and risk of developing bladder cancer, and designed with enough statistical power to permit detailed analyses and to detect smaller effects, it has several limitations which should be considered. Not all studies had information on some food items that are consumed in the Western diet, including refined grains, and potatoes. Including these items might help to better examine the association between the Western dietary pattern diet and bladder cancer. However, these factors were not fully considered as main components of the Western dietary pattern by previous studies.^21^, ^57^ It worth noting that as the definition of a Western diet may vary between different studies,^43^, ^57^, ^58^ by conducting a comprehensive review on the literature we used a more common definition of Western diet to create a Western diet adherence score.^14^, ^15^, ^16^, ^17^ Also, limited information was available for some possible risk factors of bladder cancer, such as body mass index, physical inactivity, socioeconomic status and occupational exposures to carcinogenic chemicals. The possibility to adjust for these factors would have allowed more accurate risk estimates. Although, the current literature suggests only a small proportion of bladder cancer cases can be attributed to these factors.^5^, ^59^, ^60^ We were also not able to take into account any possible changes to dietary and lifestyle habits over time, which would better reflect the effect of long‐term diet. Likewise, information bias, which as a consequence of self‐reported information on food consumption is a common bias in nutritional epidemiology studies,^61^ should be taken into account when intenerating results. However, it is expected that the distribution of this bias was not significantly different between cases and noncases, suggesting that the impact of information bias on our findings might be minimal.

## 5 CONCLUSIONS

In conclusion, our analysis revealed that higher adherence to a Western dietary pattern is associated with increased risk of bladder cancer, particularly for men. This finding supports the hypothesis that Western dietary pattern may play a role in the etiology of bladder cancer. Further research is necessary to investigate the possible mechanisms for the Western dietary pattern effects on carcinogenesis of bladder cancer and to identify the components of Western dietary pattern that may be predominantly responsible for the observed association with bladder cancer risk.

## CONFLICT OF INTEREST

The authors declare that they have no conflict of interest.

6

## ETHICS STATEMENT

Each participating study has been approved by the local ethics committee. Informed consent was obtained from all individual participants included in each study.

## Supporting information


**Appendix S1** Supporting information

## Data Availability

Datasets that are minimally required to replicate the outcomes of the study will be made available upon reasonable request.
