# Supplementary material for: Adherence to a Western dietary pattern and risk of bladder cancer: A pooled analysis of 13 cohort studies of the Bladder Cancer Epidemiology and Nutritional Determinants international study
Source: Int J Cancer. 2020 Jul 20;147(12):3394–403. doi: 10.1002/ijc.33173 (PMC7689707; doi:10.1002/ijc.33173)
Supplement: Supplementary file 1 — Appendix S1 Supporting information [file IJC-147-3394-s001.pdf]

Supplementary Material

Adherence to a Western dietary pattern and risk of bladder cancer: a pooled analysis of 13 Cohort Studies of the Bladder Cancer Epidemiology and Nutritional Determinants (BLEND) International Study

Mostafa Dianatinasab, Anke Wesselius, Amin Salehi-Abargouei, Evan Y.W. Yu, Maree Brinkman, Mohammad Fararouei, Piet van den Brandt, Emily White, Elisabete Weiderpass, Florence Le Calvez-Kelm, Marc Gunter, Inge Huybrechts, Fredrik Liedberg, Guri Skeie, Anne Tjonneland, Elio Riboli, Graham G. Giles, Roger L. Milne, Maurice P. Zeegers

Supplementary Table 1. Hazard ration (HR) and 95% confidence intervals (CIs) based on tertile of Western diet score stratified by gender and smoking status.

Supplementary Table 2. Hazard ration (HR) and 95% confidence intervals (CIs) based on tertile of Western diet score by cancer sub-types.

Supplementary Table 1. Hazard ration (HR) and 95% confidence intervals (CIs) based on tertile of Western diet score stratified by gender and smoking status.

Subgroup	Tertile 1<br>HR (95%CI) *	Tertile 2<br>HR (95%CI)	Tertile 3<br>HR (95%CI)	*P trend*
**Women**				
**Current smoker**				-
**Participants** (*number*)<br>Case/non-case	56/ 23,263	94/ 23,912	88/ 23,674	-
Pearson year	248664.7	265448.4	266072.3	-
Crude	1 (reference)	1.38 (0.99, 1.91)	1.25 (0.90, 1.75)	0.217
Model 1 [1]	1 (reference)	1.45 (1.03, 0.03)	1.40 (0.97, 2.02)	0.080
Model 2 [2]	1 (reference)	1.45 (1.03, 2.03)	1.36 (0.96, 2.00)	0.999
**Former smoker**				-
**Participants** (*number*)<br>Case/non-case	86/ 35,031	94/ 32,064	47/ 26,387	-
Pearson year	357183.6	363339.4	299911.1	-
Crude	1 (reference)	1.02 (0.76, 1.37)	0.70 (0.49, 1.00)	0.079
Model 1 [1]	1 (reference)	1.10 (0.81, 1.49)	0.80 (0.54, 1.19)	0.377
Model 2 [2]	1 (reference)	1.11 (0.81, 1.51)	0.81 (0.55, 1.21)	0.425
**Never smoker**				
**Participants** (*number*)<br>Case/non-case	114/ 82,364	154/ 76,505	87/ 64, 055	-
Pearson year	903011.4	890789.3	732229.1	-
Crude	1 (reference)	1.38 (1.09, 1.77)	1.09 (0.82, 1.45)	0.358
Model 1 [1]	1 (reference)	1.49 (1.16, 1.91)	1.28 (0.95, 1.47)	0.055
Model 2 [2]	1 (reference)	1.47 (1.14, 1.89)	1.27 (0.93, 1.72)	0.073
**Men**				
**Current smoker**				
**Participants** (*number*)<br>Case/non-case	166/ 9,873	364/ 14,974	465/ 22,358	-
Pearson year	106860.5	170458	252179	-
Crude	1 (reference)	1.49 (1.24, 1.80)	1.47 (1.23, 1.76)	<0.001
Model 1 [1]	1 (reference)	1.56 (1.29, 1.88)	1.61 (1.34, 1.95)	0.001
Model 2 [2]	1 (reference)	1.63 (1.35, 1.96)	1.78 (1.46, 2.16)	<0.001
**Former smoker**				
**Participants** (*number*)<br>Case/non-case	305/ 25,328	469/ 24,844	461/ 26,312	-
Pearson year	243256	282458.2	308673.5	-
Crude	1 (reference)	1.40 (1.21, 1.62)	1.55 (1.34, 1.80)	<0.001
Model 1 [1]	1 (reference)	1.47 (1.26, 1.70)	1.71 (1.46, 2.00)	0.001
Model 2 [2]	1 (reference)	1.44 (1.24, 1.68)	1.67 (1.42, 1.96)	<0.001
**Never smoker**				
**Participants** (*number*)<br>Case/non-case	93/ 22,394	140/ 22,524	116/ 21,505	-
Pearson year	227755.3	270656.7	262359.7	-
Crude	1 (reference)	1.38 (1.06, 1.79)	1.35 (1.02, 1.78)	0.032
Model 1 [1]	1 (reference)	1.40 (1.07, 1.84)	1.39 (1.04, 1.87)	0.029

Model 2 [2]	1 (reference)	1.39 (1.06, 1.83)	1.37 (1.01, 1.85)	0.045

[*] HR= hazard ratio, CI= confidence interval.

[1] adjusted for energy intake, smoking status, smoking intensity, age and sex.

[2] adjusted for model 1+ fluid intake, fruit and vegetables intakes.

Supplementary Table 2. Hazard ration (HR) and 95% confidence intervals (CIs) based on tertile of Western diet score by cancer sub-types.

	**Tertile 1** HR (95%CI) *	**Tertile 2** HR (95%CI)	**Tertile 3** HR (95%CI)	***P trend***
**NMIBC[*]***				-
**Participants** (*number)* Cases	334	547	484	-
Pearson year	2469.457	5147.161	4333.148	-
Crude	1 (reference)	0.83 (0.72, 0.95)	0.94 (0.82, 1.09)	0.701
Model 1 [1]	1 (reference)	1.10 (0.91, 1.31)	1.40 (1.16, 1.69)	<0.001
Model 2 [2]	1 (reference)	1.09 (0.86, 1.37)	1.28 (1.02, 1.63)	<0.001
**MIBC[+]**				-
**Participants** (*number)* Cases	189	380	305	-
Pearson year	1616.891	3944.66	3117.87	-
Crude	1 (reference)	0.87 (0.73, 1.04)	0.93 (0.78, 1.12)	0.672
Model 1 [1]	1 (reference)	1.20 (0.95, 1.50)	1.33 (1.05, 1.69)	0.019
Model 2 [2]	1 (reference)	1.09 (0.86, 1.37)	1.28 (1.01, 1.64)	0.028

[*] HR= hazard ratio, CI= confidence interval. ** NMIBC = non-muscle-invasive bladder cancer, [+]MIBC= muscle-invasive bladder cancer.

[1] adjusted for energy intake, smoking status, smoking intensity, age and sex.

[2] adjusted for model 1+ fluid intake, fruit and vegetables intakes.
